# Allele frequency of variants reported to cause adenine phosphoribosyltransferase deficiency

**DOI:** 10.1038/s41431-020-00805-6

**Published:** 2021-03-11

**Authors:** Hrafnhildur L. Runolfsdottir, John A. Sayer, Olafur S. Indridason, Vidar O. Edvardsson, Brynjar O. Jensson, Gudny A. Arnadottir, Sigurjon A. Gudjonsson, Run Fridriksdottir, Hildigunnur Katrinardottir, Daniel Gudbjartsson, Unnur Thorsteinsdottir, Patrick Sulem, Kari Stefansson, Runolfur Palsson

**Affiliations:** 1 Faculty of Medicine, School of Health Sciences, University of Iceland, Reykjavik, Iceland; 2 Internal Medicine and Rehabilitation Services, Landspitali—The National University Hospital, Reykjavik, Iceland; 3 Renal Unit, Newcastle upon Tyne Hospitals NHS Foundation Trust, Newcastle upon Tyne, UK; 4 Institute of Genetic Medicine, Newcastle University, Central Parkway Newcastle, Newcastle upon Tyne, UK; 5 NIHR Biomedical Research Centre, Newcastle upon Tyne, UK; 6 Children’s Medical Center, Landspitali—The National University Hospital, Reykjavik, Iceland; 7 deCODE genetics, Reykjavik, Iceland; 8 School of Engineering and Natural Sciences, University of Iceland, Reykjavik, Iceland

**Keywords:** Genomics, Medical research

## Abstract

Adenine phosphoribosyltransferase deficiency is a rare, autosomal recessive disorder of purine metabolism that causes nephrolithiasis and progressive chronic kidney disease. The small number of reported cases indicates an extremely low prevalence, although it has been suggested that missed diagnoses may play a role. We assessed the prevalence of APRT deficiency based on the frequency of causally-related *APRT* sequence variants in a diverse set of large genomic databases. A thorough search was carried out for all *APRT* variants that have been confirmed as pathogenic under recessive mode of inheritance, and the frequency of the identified variants examined in six population genomic databases: the deCODE genetics database, the UK Biobank, the 100,000 Genomes Project, the Genome Aggregation Database, the Human Genetic Variation Database and the Korean Variant Archive. The estimated frequency of homozygous genotypes was calculated using the Hardy-Weinberg equation. Sixty-two pathogenic *APRT* variants were identified, including six novel variants. Most common were the missense variants c.407T>C (p.(Met136Thr)) in Japan and c.194A>T (p.(Asp65Val)) in Iceland, as well as the splice-site variant c.400 + 2dup (p.(Ala108Glufs*3)) in the European population. Twenty-nine variants were detected in at least one of the six genomic databases. The highest cumulative minor allele frequency (cMAF) of pathogenic variants outside of Japan and Iceland was observed in the Irish population (0.2%), though no APRT deficiency cases have been reported in Ireland. The large number of cases in Japan and Iceland is consistent with a founder effect in these populations. There is no evidence for widespread underdiagnosis based on the current analysis.

## Introduction

Adenine phosphoribosyltransferase (APRT) deficiency (OMIM 102600; 614723) is a rare disorder of purine metabolism that is inherited in an autosomal recessive manner [1]. In the absence of functional APRT activity, adenine is oxidized to 2,8-dihydroxyadenine (DHA) by xanthine oxidoreductase (xanthine dehydrogenase/oxidase). Poor solubility of DHA in the urine leads to stone formation and crystal nephropathy. Radiolucent kidney stones are the most common clinical manifestation, followed by chronic kidney disease which has already progressed to end-stage kidney disease in 15–20% of patients at the time of diagnosis of APRT deficiency [2]. In some cases, the disorder is first recognized in the setting of disease recurrence following kidney transplantation. Treatment with the xanthine oxidoreductase inhibitors allopurinol and febuxostat reduces DHA synthesis and excretion, alleviating stone burden and kidney injury [1].

The human *APRT* gene, located on chromosome 16q24, is 2466 base pairs long and contains five exons encoding a 180 amino acid protein (NP_000476.1) [3]. The Human Gene Mutation Database (HGMD) lists 51 reported disease-causing variants to date. More than 300 cases have been reported worldwide, the majority of patients coming from Japan, France and Iceland [1]. The most commonly reported variants are a missense variant in exon 3, c.407T>C (p.(Met136Thr)), in patients from Japan [4, 5], a T insertion at the splice donor site of intron 4, c.400 + 2dup (p.(Ala108Glufs*3)), among Europeans [6, 7], and another missense variant in exon 3, c.194A>T (p.(Asp65Val)), which accounts for all known cases of APRT deficiency in Iceland [8, 9]. Individuals homozygous for disease-causing variants have invariably been shown to have completely abolished enzyme activity, while heterozygous carriers have partial enzyme function but do not appear to have any biochemical or clinical abnormalities [10].

Although the small number of reported cases worldwide indicates that APRT deficiency is an extremely rare condition, the large number of patients identified in Japan, France and Iceland has raised concerns that the prevalence may be underestimated in many populations. Furthermore, the commonly reported occurrence of delayed or missed diagnosis suggests that lack of awareness among clinicians might contribute to the low number of reported cases [1, 10, 11]. However, a founder effect is a likely explanation for the high frequency of a single pathogenic variant and disease prevalence in the Icelandic [9, 12] and Japanese populations [5, 13].

As whole-genome and whole-exome sequencing have become more widely available, studies on the molecular basis of rare diseases using open-access population-based data have provided further insight into their pathogenesis and allele frequency [14–16]. The aim of this study was to search for all *APRT* variants confirmed as disease-causing under recessive mode of inheritance that have been reported in the literature and registered in online disease databases to date, and assess the frequency of these variants in a set of large population genomic databases containing information on individuals from diverse geographic locations and ethnic groups.

## Methods

### Ethical considerations

The study was approved by the National Bioethics Committee of Iceland (NBC 09-072) and the Icelandic Data Protection Authority.

### Search for known pathogenic *APRT* sequence variants

In order to identify all sequence variants documented to cause APRT deficiency, we performed a search strategy using three major sources through February 2020:

1. Medical literature and databases. We performed a web-based search and assessement of *APRT* variants in reported cases of APRT deficiency using PubMed, HGMD [17], OMIM [18], and ClinVar [19].
2. Expanded web sources. To search for *APRT* variants, we also assessed the full text of published articles, conference abstracts and book chapters identified using PubMed® (https://pubmed.ncbi.nlm.nih.gov/) and Google® (http://www.google.com), with the terms “APRT deficiency”, “adenine phosphoribosyltransferase deficiency”, “2,8-dihydroxyadenine”, “2,8-dihydroxyadeninuria” and “2,8-DHA”.
3. APRT Deficiency Registry of the Rare Kidney Stone Consortium (RKSC; http://www.rarekidneystones.org). Information on *APRT* variants was obtained from the APRT Deficiency Registry and through personal communication to the research group. The RKSC was established in 2009 to study hereditary kidney stone disorders and has since maintained a private database with clinical information on participating individuals. As of September 2019, 63 patients from eight countries were enrolled in the APRT Deficiency Registry, 56 of whom had undergone genetic testing with the identification of biallelic disease-causing *APRT* variants.

The variants are reported using nomenclature recommended by the Human Genome Variation Society, including reference sequence numbers (NM_000485.2 and NG_008013.1) and transcript number (ENST00000426324.6).

As consequence at the RNA level has not been reported for the vast majority of the variants, a prediction analysis of the variant effect at the splice junction on the splicing was carried out using consensus sequence frequencies, maximum entropy modeling and the varSEAK Online splice prediction tool (https://varseak.bio/).

### Search for known pathogenic *APRT* variants in genomic databases

We looked for the pathogenic variants, identified using the search strategy described above, in public whole-genome and whole-exome databases through February 2020. The individual and cumulative frequency of these disease-causing variants was determined.

We used six databases in all, containing information on sequence variation for over 300,000 individuals from various geographic locations and ethnic groups:

1. The database at deCODE genetics [20] comprises whole-genome sequencing data obtained from 53,964 Icelanders, 3153 Swedes, 8831 Danes, 2920 Norwegians and 1365 Irish persons enrolled in various studies.
2. The UK Biobank Project [21], a large prospective cohort study, includes exome sequencing data on approximately 50,000 individuals (by 2019).
3. The 100,000 Genomes Project [22] contains data on whole-genome sequencing of 63,737 patients with rare diseases from across the UK.
4. The Genome Aggregation Database (gnomAD) [23] browser (version 2.1.1) includes whole-genome and whole-exome sequencing data on 141,456 unrelated individuals sequenced as a part of various disease-specific and population genetic studies of diverse countries and ethnicities. The database includes whole-exome sequencing of 8128 Africans and African-Americans, 17,296 Latinos, 5040 Ashkenazi Jews, 9197 East Asians, 10,824 Finnish and 56,885 non-Finnish Europeans, 15,308 South Asians and 3070 individuals classified as “Other“.
5. The Human Genetic Variation Database of Japan [24, 25] comprises exome sequencing data on 1208 individuals and genotyping information for common variations from 3248.
6. The Korean Variant Archive (KOVA) [26] includes exome sequencing data on 1055 healthy Korean individuals.

The minor allele frequency (MAF) of each causally-related sequence variant among different ancestries was exctracted.

### Determination of cumulative allele frequency of known pathogenic *APRT* variants and estimation of homozygous genotype frequency

To determine the cumulative minor allele frequency (cMAF) of the reported *APRT* variants, we used the sum of the allele frequency of individual variants in databases of genome and exome sequences available for a given geographic area or ethnic group. We note that each of the individual variants is rare and none are reported to be present on the same haplotype. As APRT deficiency is an autosomal recessive disease, each affected individual is expected to carry two copies of a disease-causing variant, either the same (homozygous) or different variants (compound heterozygous). The Hardy-Weinberg equation (*p*^2^ + 2*pq* + *q*^2^ = 1) was used to calculate the expected genotype frequencies for heterozygous (2*pq*) and homozygous (*p*^2^) genotypes, using the minor and total allele counts, where p is equal to the cMAF of causally-related variants and *q* is (1 − *p*).

## Results

### Pathogenic *APRT* variants

Using a comprehensive search strategy, 62 pathogenic variants in the *APRT* gene were identified in homozygous or compound heterozygous patients with APRT deficiency (Supplementary Tables S1 and S2). All 62 variants were present in patients with clinical findings characteristic of APRT deficiency and/or abolished APRT enzyme function (94% of variants). In total, 482 cases of APRT deficiency from 33 countries have been reported worldwide, of which 311 (64.5%) have received a molecular diagnosis to the best of our knowledge. Thirty-seven variants were each detected in a single affected individual. The remaining 25 variants were observed in two or more cases, including ten which were found in at least five individuals. There were 28 missense variants and the remaining 35 were nonsense, insertion or deletion, frameshift and start loss, and splice variants. Of nine presumed splice variants, six were predicted to have a splicing effect and one a likely splicing effect (Supplementary Table S3).

We also noted two sequence variants that we do not classify as pathogenic, each of which were reported in a single heterozygous individual with partial APRT enzyme deficiency; a missense variant, c.266G>A (p.(Arg89Gln)), from Australia and the c.346G>A (p.(Ala116Thr)) missense variant from China. These variants were quite common in the databases searched; in gnomAD, the p.(Ala116Thr) variant had an allele frequency of 0.23% (46/19,942) in the East Asian population and p.(Arg89Gln) an allele frequency of 0.41% (125/30,584) in the South Asian population. However, neither of the two variants have been observed in confirmed cases of APRT deficiency and, thus, were not included in our set of 62 causally-related variants.

Of the 62 pathogenic *APRT* variants, 56 had already been reported in the literature. The missense variants c.407T>C (p.(Met136Thr)) and c.194A>T (p.(Asp65Val)) and the splice-site variant c.400 + 2dup (p.(Ala108Glufs*3)) were most commonly reported. Additionally, six novel pathogenic variants were discovered through our APRT Deficiency Research Program. A C-to-G substitution in intron 1 (c.81-3C>G) was identified in the homozygous state in two affected siblings in the US and one patient in Italy, and a C-to-T substitution in exon 1 (c.58C>T; p.(Pro20Ser)) was detected in a homozygous patient in the UK. Two compound heterozygous patients from the US had novel sequence variants; one of these patients had a frameshift variant in exon 1, c.23dup (p.(Val9Glyfs*2)), and a deletion in exon 5, c.522_524del (p.(Ser175del)), while the other had a missense variant in exon 3, c.264G>T (p.(Lys88Asn)), in addition to a variant that had already been reported. The sixth variant was identified in a patient from India who was referred to our program but had already been found to be a compound heterozygote when evaluated in his home institution, carrying a missense variant in exon 3, c.227C>T (p.(Ala76Val)), in addition to one previously reported variant.

### The three most common pathogenic *APRT* variants

All cases harboring the three most common variants causing APRT deficiency in a homozygous or compound heterozygous state are presented in Table 1. For these three variants, we summarized the counts of reported cases and the frequencies of these variants in different geographic locations and ethnic groups represented in the large population genomic databases. These three variants were observed in 368 (63.8%) of the 576 of causally-related alleles detected in the APRT deficiency cases.

**Table 1:** The frequency of the three most common pathogenic *APRT* variants among all identified cases worldwide and in population genomic databases.

	Identified cases		Genomic databases		
	Homozygotes	Compound heterozygotes	Allele count	Allele number	Allele frequency (%)
**c.407T>C (p.(Met136Thr))**					
Japan	89	44			
Japan (HGVD)			3	2326	0.13
USA	1				
Korea (KOVA)			1	1898	0.05
*gnomAD*					
African			0	16,760	0.000
Ashkenazi Jewish			0	9956	0.000
East Asian			1	18,360	0.005447
European (Finnish)			0	20,398	0.000
European (Non-Finnish)			0	112,090	0.000
Latino			0	34,518	0.000
Other			0	6066	0.000
South Asian			0	30,590	0.000
**c.194A>T (p.(Asp65Val))**					
Iceland	37				
Iceland (deCODE)			1299	107,928	1.2
Sweden (deCODE)			1	6306	0.016
Denmark (deCODE)			1	17,662	0.006
UK	1				
UK (100,000 Genomes Project)			1	127,474	0.0008
Spain	1				
France		1			
Australia		1			
*gnomAD*					
African			0	24,072	0.000
Ashkenazi Jewish			0	10,156	0.000
East Asian			0	19,736	0.000
European (Finnish)			0	24,188	0.000
European (Non-Finnish)			1	124,598	0.0008026
Estonian			1	4768	0.02097
Latino			0	35,102	0.000
Other			0	7032	0.000
South Asian			0	30,262	0.000
c.400 + 2dup (p.(Ala108Glufs*3))					
Europe	13	23			
UK (Biobank)			22	1,000,000	0.022
UK (100,000 Genomes Project)			4	127,474	0.003
Ireland (deCODE)			5	2730	0.18315018
Denmark (deCODE)			4	17,662	0.02264749
USA	6				
Australia		1			
Unknown	1				
*gnomAD*					
African			1	24,940	0.004010
Ashkenazi Jewish			0	10,350	0.000
East Asian			0	19,942	0.000
European (Finnish)			0	24,890	0.000
European (Non-Finnish)			27	128,838	0.02096
South European			5	11,592	0.04313
Other non-Finnish European			10	32,948	0.03035
North-West European			11	50,708	0.02169
Swedish			1	26,100	0.003831
Latino			1	35,430	0.002822
Other			0	7280	0.000
South Asian			1	30,614	0.003266

*gnomAD* Genome Aggregation Database, *HGVD* Human Genome Variation Database, *KOVA* Korean Variant Archive.

#### The missense variant, c.407T>C (p.(Met136Thr))

The T-to-C missense variant at codon 136 in exon 5, p.(Met136Thr), has been described in 134 patients of Japanese descent, all but one of whom were previously reported in the literature. The additional case, identified in the APRT Deficiency Registry, was a Japanese patient living in the US. This pathogenic variant was also found in the Human Genetic Variation Database in Japanese cohorts from Tohoku University (MAF 1.3%, Alt/Ref 1/76) and the University of Tokyo (MAF 0.3%, Alt/Ref 2/666). This geographic distribution is consistent with previous reports [13]. In KOVA, the MAF of this variant was 0.05% (1/1898). The variant was not detected in other genomic databases. We note that the number of Japanese in the gnomAD database is very low, only 150.

#### The missense variant, c.194A>T (p.(Asp65Val))

Forty-one cases of APRT deficiency carrying the p.(Asp65Val) variant have been reported. Of these, 37 individuals are from Iceland, all of whom are homozygous. One patient from Spain and one from the UK were homozygous for the same variant and two other patients were compound heterozygotes, one from France and one from Australia. On whole-genome sequencing of 53,964 Icelanders in the deCODE database, we observed this *APRT* variant in 1299 alleles, yielding a minor allele frequency (MAF) of 1.2% (1299/107,928). Icelanders have ancestries originating from Scandinavia and the British Islands. Out of 14,904 whole-genome sequenced Scandinavian individuals at deCODE, only two heterozygotes carrying the p.(Asp65Val) variant were detected, and one single heterozygote was identified in the UK (Supplementary Table S4).

#### The splice-site variant, c.400 + 2dup (p.(Ala108Glufs*3))

The c.400 + 2dup variant was found in a total of 41 cases that all are of European descent. This variant has been identified in 13 homozygotes and 23 compound heterozygotes from Europe, including France (*n* = 29), Germany, Austria, Belgium, Italy and Poland. The variant has also been found in patients in the US (*n* = 6) and Australia (n = 1). The variant was consistently present in European populations in the genomic databases (Supplementary Tables S4–S6). The highest frequency was observed among Irish (MAF 0.18%; 5/2730) and Southern European (MAF 0.043%; 5/11,592) individuals.

### Allele frequency of the pathogenic *APRT* variants in population databases

We searched for the presence and assessed the frequency of the 62 pathogenic *APRT* variants within the publicly available databases, by geographic location and ethnicity. Out of the 62 variants, we observed 29 in the population databases.

Two of the three most common variants described above, p.(Met136Thr) and p.(Asp65Val), are clearly indicative of a founder effect in Japan and Iceland, respectively, with very low frequency in the public databases. The third variant, c.400 + 2dup, shows a wide distribution among cases from multiple European countries and the US, and is the disease-causing variant with the highest occurence in public databases. Besides these three variants, the most commonly reported ones were c.294G>A (p.(Trp98*)) in 26 Japanese cases and the c.521_523del (p.(Phe174del)) variant which was observed in six cases from European countries and three in the US.

Iceland is the country where the highest proportion of the population has been sequenced (around one in six Icelanders). In addition to the p.(Asp65Val) variant, we discovered five individuals heterozygous for one of the two other known pathogenic *APRT* variants in the deCODE database (Table 2). Of these, the c.1A>C (p.(Met1?)) nonsense variant, previously reported in patients from Hungary and France, was observed in four Icelanders with a MAF of 0.004% (4/107,928).

**Table 2:** Pathogenic *APRT* variants detected in the deCODE genetics database.

No.	Position (Build 38)	Location	Base change	Amino acid change	Variant type	Alt/Ref	MAF (%)	Country of origin
1	16:88810550	Exon 3	c.194A>T	p.(Asp65Val)	Missense	1299/107928	1.2	Iceland
2	16:88811899	Exon 1	c.1A>C	p.(Met1?)	Nonsense	4/107928	0.003706	Iceland
3	16:88810141	Exon 4	c.329T>C	p.(Leu110Pro)	Missense	1/107928	0.000927	Iceland
4	16:88810141	Exon 4	c.329T>C	p.(Leu110Pro)	Missense	5/17662	0.028309	Denmark
5	16:88810067	Intron 4	c.400+2dup	p.(Ala108Glufs*3)	Indel	4/17662	0.02264749	Denmark
6	16:88811899	Exon 1	c.1A>C	p.(Met1?)	Nonsense	2/17662	0.01132374	Denmark
7	16:88810550	Exon 3	c.194A>T	p.(Asp65Val)	Missense	1/17662	0.00566187	Denmark
8	16:88811899	Exon 1	c.1A>C	p.(Met1?)	Nonsense	1/6306	0.015857913	Sweden
9	16:88810550	Exon 3	c.194A>T	p.(Asp65Val)	Missense	1/6306	0.015857913	Sweden
10	16:88809717	Exon 5	c.521_523del	p.(Phe174del)	Deletion	1/6306	0.015857913	Sweden
11	16:88810067	Intron 4	c.400 + 2dup	p.(Ala108Glufs*3)	Indel	5/2730	0.18315018	Ireland
12	16:88809700	Exon 5	c.541T>C	p.(*181Argext*?)	Stop lost	2/2730	0.07326007	Ireland
13	16:88810494	Exon 3	c.250G>A	p.(Val84Met)	Missense	2/5840	0.0342465	Norway
14	16:88809717	Exon 5	c.521_523del	p.(Phe174del)	Deletion	1/5840	0.0171232876	Norway

Reference sequence: NG_008013.1 (NM_000485.2).

*Alt/ref* alternate/reference alleles, *Indel* insertion or deletion, *MAF* minor allele frequency.

### Estimation of homozygous *APRT* genotype frequency

Based on the Hardy-Weinberg principle, the cMAF of p.(Asp65Val) in the Icelandic population was 1.2%, with a predicted frequency of homozygous individuals of 1 in 6840 (Table 3). In the Scandinavian countries (Denmark, Norway and Sweden), the cumulative allele frequency of any of the pathogenic variants in 14,904 individuals was similar among the three nations, or around 0.05%. This would correspond to an expected number of homozygotes of about one in four million individuals, or five cases in a total population of 20 million people. We note that no cases of APRT deficiency have been reported in these three countries. In Ireland, the cMAF of the pathogenic variants observed was 0.26% (7/2730 alleles), represented by five copies of c.400 + 2dup and a single copy of two other disease-causing variants. The expected frequency of Irish individuals homozygous for a pathogenic variant is 1 in 152,100, corresponding to roughly 30 homozygous subjects given the size of the Irish nation.

**Table 3:** Cumulative allele count, allele frequency and expected homozgous frequency of pathogenic *APRT* variants observed in population genomic databases.

	Allele count (Alt/Ref)	Allele frequency (%)	Expected frequency of homozygotes (1 in)	Expected carrier frequency (1 in)
**gnomAD**				
African	9/24,940	0.0360	7,679,057	1386
Ashkenazi Jewish	1/10,350	0.0097		
East Asian	9/19,946	0.0451	4,911,641	1109
European (Finnish)	1/24,920	0.0080		
European (Non-Finnish)	63/128,828	0.0489	4,181,571	1023
Latino	11/35,430	0.0508	3,712,044	964
South Asian	5/30,614	0.0163	37,488,680	3062
**UK Biobank**				
All	77/1,000,000	0.071	1,686,625	650
**100,000 Genomes Project**				
All	11/127,474	0.0086	134,294,386	5795
**deCODE**				
Iceland	1304/107,928	1.2082	6840	42
Denmark	12/17,662	0.0678	2,166,293	736
Sweden	3/6306	0.0476	4,418,404	1052
Norway	3/5840	0.0514	3,789,511	974
Ireland	7/2730	0.18315	298,116	274
**HGVD**	3/2326	0.1289	601,142	388
**KOVA**	1/1898	0.052687	3,602,404	950

*gnomAD* Genome Aggregation Database, *HGVD* Human Genome Variation Database, *KOVA* Korean Variant Archive.

The overall frequency of the reported pathogenic variants in gnomAD was similar among the European (non-Finnish) population, with a calculated allele frequency of 0.05% (63/128,838). Frequencies of causally-related variants in Latinos and East Asians was similar to that observed in gnomAD for Europeans, whereas other groups had lower frequencies. Thus, there could be as many as 300 cases in the East Asian population (1.7 billion) and up to 170 cases in the Latin American population (642 million).

## Discussion

A comprehensive search for APRT deficiency cases, using multiple web-based resources and the APRT Deficiency Registry, identified 62 pathogenic *APRT* variants in a total of 311 patients undergoing molecular diagnosis. Six novel variants were discovered through the APRT Deficiency Registry. Three variants are by far most common among the APRT deficiency cases, namely the missense variants p.(Met136Thr) and p.(Asp65Val) and the splice variant c.400 + 2dup, collectively accounting for 64% of the disease-associated alleles.

Twenty-nine of the 62 pathogenic variants were detected in large population genomic databases and were assessed for the individual and cumulative frequencies. Evaluation of the frequency of the Icelandic missense variant p.(Asp65Val) in large datasets from ancestral populations in Scandinavia and UK revealed that the variant is approximately 100 times less common in these geographic locations. Thus, the high carrier rate of the variant of 1.2% in the Icelandic population clearly represents a founder effect. Not surprisingly, analysis of the large Icelandic genomic data disclosed a number of additional undiagnosed homozygous individuals.

The high frequency of the missense variant p.(Met136Thr) in the Japanese population is also consistent with a founder effect. A previous study assessing the frequency of p.(Met136Thr) observed an allele frequency of 0.13% (3/2326) among Japanese and 0.05% (1/1898) among Koreans, while this variant was absent in samples from Taiwanese individuals. In the same study, the geographic distribution in Japan was determined to be rather uniform [13].

The c.400 + 2dup variant was observed in cases from many countries in Europe, the US and Australia, as well as in most of the public databases used in the current study. In a report of a French APRT deficiency cohort, this variant was found in 54% of the patient population, all of whom originated from metropolitan France except for one Italian family [27]. The allele frequency of the c.400 + 2dup variant was 40% in the case series. The same group of investigators also detected an allele frequency of 0.98% (2/204) by newborn screening [27].

When calculating the cMAF of reported causally-related variants to assess the expected genotypic frequency, we found that there may be as many as 200 individuals in Japan homozygous for p.(Met136Thr) and 50 in Iceland homozygous for p.(Asp65Val). As expected, the two described founder variants in Japan and Iceland are very rare outside of their respective countries. In the European (non-Finnish) population, the cMAF of any pathogenic variant was 0.05%. The cMAF in the Irish population was higher and is consistent with approximately 30 homozygous individuals. Interestingly, no cases have been diagnosed in Ireland to the best of our knowledge, indicating that the disease may be underreported or underdiagnosed.

Two *APRT* missense variants, c.266G>A (p.(Arg89Gln)) [10] and c. 346G>A (p.(Ala116Thr)) [28], have been reported in individuals with decreased enzyme function that is comparable to the heterozygous carrier state of other pathogenic *APRT* variants. It is unclear if and to what extent individuals homozygous for these variants will develop clinical manifestations of APRT deficiency. Multiple copies of both variants were present in the genomic databases and the allele frequencies were quite high in both the East Asian and South Asian populations. If proven to be disease-causing, there could be roughly 20,000 homozygotes in South Asia and 5000 in East Asia, assuming the Hardy-Weinberg equilibrium. However, as no cases of APRT deficiency caused by these two variants have been reported, a classical phenotype appears unlikely as the disease would then be seriously underdiagnosed. Hence, homozygosity for these variants would either be expected to cause a very mild clinical phenotype or no disease at all.

Of the 62 pathogenic variants, 33 were not observed in any of the public genomic databases. Most were detected in one or two cases except for c.188G>A (p.(Gly63Asp)) which was found in six patients, most of whom were of Lebanese decent. The reason for this may be the high proportion of consanguineous marriages in Lebanon, previously reported to be ~35%, and/or that Middle Eastern populations might be underrepresented in the genomic databases used in this study [29].

We also examined sequence variants that have not been detected in patients with APRT deficiency in gnomAD, which is the largest population database with the highest allele count (Supplementary Table S7). Notably, the c.362A>G (p.(Gln121Arg)) variant was quite common among the African population in gnomAD, with a MAF of 0.5% and two homozygous individuals. The confirmation of this variant or any other uncharacterized novel variants as disease-causing in patients with APRT deficiency would require a functional study.

The recent introduction of high-throughput sequencing technologies and advances in bioinformatics for variant assessment have facilitated studies of the epidemiology of monogenic diseases. Exome sequencing has led to the discovery of many novel causal genes in rare diseases [30–33]. Moreover, previously unknown pathogenic variants have been identified and undiagnosed cases have been solved. Creation of large public databases of human genomic information has generated possibilities to estimate genotypic frequencies in various populations. Thus, the allele frequency of rare causally-related variants can be assessed in multiple populations like in the current study.

The prevalence of a monogenic disease depends on the frequency of all the pathogenic variants in a population. The allele frequency of each variant can vary in different populations and minor alleles with much higher frequencies than would be expected for a rare disorder should be carefully interpreted. Correct annotation of genetic variants is important in clinical practice as misinterpretation may lead to incorrect diagnoses or harmful and ineffective treatments. A limited number of variants will generally be encountered within a given population with close to random mating. By contrast, the occurrence of the disease in inbred populations depends on the mating choice and each causally-related variant can be extremely rare, sometimes confined to a single family.

The present study has several limitations that are noteworthy. The data collection relied largely on published reports and is therefore probably incomplete. Although the reports were carefully examined, there is always a possibility that the cases were miscounted or missed. Furthermore, homozygous status in consanguinous unions, that are common in certain countries, is likely to result in underestimation of the disease prevalence in a population under study. It should be noted that publicly available genomic datasets are still scarce and the inclusion of certain ethnic groups is very limited or nonexistent. Although the gnomAD database contains data from various genomic projects in the US, the American population is not identifiable in the database so that allele frequencies cannot be assessed. Additional work is therefore required in order to characterize the prevalence of APRT deficiency around the globe. Studies of certain ethnic groups would also be of particular interest, for example the South Asian and African populations. In the future, genomic information from high-risk groups, namely patients with nephrolithiasis and/or unexplained chronic kidney disease presenting at a relatively young age, might become publicly available. Finally, the interpretation of previously unknown sequence variants remains challenging and APRT enzyme activity testing among individuals homozygous for such variants would be of interest.

In conclusion, we identified 62 confirmed pathogenic *APRT* variants, including six variants that have not been reported previously. Three of the 62 variants account for the majority of cases, two of which are mostly confined to single countries, Japan and Iceland. The high carrier rate of the missense variant p.(Asp65Val) in the Icelandic population clearly represents a founder effect and the same appears to hold true for the missense variant p.(Met136Thr) in Japan. While there is no definite indication of extensive underdiagnosis, this may nevertheless be the case in certain areas. It is important to recognize that genomic data are still lacking for many countries. Hence, future work should assess the frequency of pathogenic *APRT* variants in countries and populations where the number of expected cases is higher than currently reported. An updated list of all variants causally related to APRT deficiency will be maintained and made publicly available on the RKSC website.

## Supplementary information

Supplementary_Tables_1_To_7_Clean

## Data Availability

The pathogenic variants were submitted to ClinVar and are available at https://www.ncbi.nlm.nih.gov/clinvar/; accession numbers SCV001449056–SCV001449116.

## References

[1] Edvardsson VO, Sahota A, Palsson R. (2012) Adenine phosphoribosyltransferase deficiency. 30 Aug 2012 [Updated 2019 Sep 26]. In: Adam MP, Ardinger HH, Pagon RA, Wallace SE, Bean LJH, Stephens K, Amemiya A, editors. GeneReviews^®^ [Internet]. Seattle (WA): University of Washington, Seattle; 1993–2019. https://www.ncbi.nlm.nih.gov/books/NBK100238/. 22934314

[2] Runolfsdottir HL, Palsson R, Agustsdottir IM, Indridason OS, Edvardsson VO (2016). Kidney disease in adenine phosphoribosyltransferase deficiency. Am J Kidney Dis.

[3] Broderick TP, Schaff DA, Bertino AM, Dush MK, Tischfield JA, Stambrook PJ (1987). Comparative anatomy of the human APRT gene and enzyme: nucleotide sequence divergence and conservation of a nonrandom CpG dinucleotide arrangement. Proc Natl Acad Sci USA..

[4] Hidaka Y, Tarle SA, Fujimori S, Kamatani N, Kelley WN, Palella TD (1988). Human adenine phosphoribosyltransferase deficiency. Demonstration of a single mutant allele common to the Japanese. J Clin Investig..

[5] Kamatani N, Hakoda M, Otsuka S, Yoshikawa H, Kashiwazaki S (1992). Only three mutations account for almost all defective alleles causing adenine phosphoribosyltransferase deficiency in Japanese patients. J Clin Investig..

[6] Gathof BS, Sahota A, Gresser U, Chen J, Stambrook PJ, Tischfield JA (1990). Identification of a splice mutation at the adenine phosphoribosyltransferase locus in a German family. Klin Wochenschr..

[7] Bollee G, Dollinger C, Boutaud L, Guillemot D, Bensman A, Harambat J (2010). Phenotype and genotype characterization of adenine phosphoribosyltransferase deficiency. J Am Soc Nephrol..

[8] Chen J, Sahota A, Laxdal T, Scrine M, Bowman S, Cui C (1991). Identification of a single missense mutation in the adenine phosphoribosyltransferase (APRT) gene from five Icelandic patients and a British patient. Am J Hum Genet..

[9] Edvardsson V, Palsson R, Olafsson I, Hjaltadottir G, Laxdal T (2001). Clinical features and genotype of adenine phosphoribosyltransferase deficiency in Iceland. Am J Kidney Dis..

[10] Sahota AS, Tischfield JA, Kamatani N, Simmonds HA. Adenine phosphoribosyltransferase deficiency and 2,8-dihydroxyadenine lithiasis. In: Scriver CR, Beaudet AL, Sly WS, Valle D, editors; Childs B, Kinzler KW, Vogelstein B, associate editors. The Metabolic and Molecular Bases of Inherited Disease. Vol 1. 8th ed. New York, NY: McGraw-Hill; 2001:2571–84.

[11] Hidaka Y, Palella TD, O’Toole TE, Tarle SA, Kelley WN (1987). Human adenine phosphoribosyltransferase. Identification of allelic mutations at the nucleotide level as a cause of complete deficiency of the enzyme. J Clin Investig..

[12] Oddsson A, Sulem P, Helgason H, Edvardsson VO, Thorleifsson G, Sveinbjornsson G (2015). Common and rare variants associated with kidney stones and biochemical traits. Nat Commun..

[13] Kamatani N, Terai C, Kim SY, Chen CL, Yamanaka H, Hakoda M (1996). The origin of the most common mutation of adenine phosphoribosyltransferase among Japanese goes back to a prehistoric era. Hum Genet..

[14] Hopp K, Cogal AG, Bergstralh EJ, Seide BM, Olson JB, Meek AM (2015). Phenotype-genotype correlations and estimated carrier frequencies of primary hyperoxaluria. J Am Soc Nephrol..

[15] Vasiljevic E, Ye Z, Pavelec DM, Darst BF, Engelman CD, Baker MW (2019). Carrier frequency estimation of Zellweger spectrum disorder using ExAC database and bioinformatics tools. Genet Med..

[16] Hanany M, Sharon D (2019). Allele frequency analysis of variants reported to cause autosomal dominant inherited retinal diseases question the involvement of 19% of genes and 10% of reported pathogenic variants. J Med Genet..

[17] Stenson PD, Ball EV, Mort M, Phillips AD, Shaw K, Cooper DN. The Human Gene Mutation Database (HGMD) and its exploitation in the fields of personalized genomics and molecular evolution. Curr Protoc Bioinform. 2012; Chapter 1:Unit 1.13. 10.1002/0471250953.bi0113s39 22948725

[18] Takeuchi H, Kaneko Y, Fujita J, Yoshida O (1993). A case of a compound heterozygote for adenine phosphoribosyltransferase deficiency (APRT*J/APRT*Q0) leading to 2,8-dihydroxyadenine urolithiasis: review of the reported cases with 2,8-dihydroxyadenine stones in Japan. J Urol..

[19] Landrum MJ, Lee JM, Benson M, Brown G, Chao C, Chitipiralla S (2016). ClinVar: public archive of interpretations of clinically relevant variants. Nucleic Acids Res..

[20] Gudbjartsson DF, Helgason H, Gudjonsson SA, Zink F, Oddson A, Gylfason A (2015). Large-scale whole-genome sequencing of the Icelandic population. Nat Genet..

[21] Sudlow C, Gallacher J, Allen N, Beral V, Burton P, Danesh J (2015). UK biobank: an open access resource for identifying the causes of a wide range of complex diseases of middle and old age. PLoS Med..

[22] Genomics England. The National Genomics Research and Healthcare Knowledgebase v5; 2019. 10.6084/m9.figshare.4530893.v5.

[23] Karczewski KJ, Francioli LC, Tiao G, Cummings BB, Alföldi J, Wang Q (2020). The mutational constraint spectrum quantified from variation in 141,456 humans. Nature.

[24] Higasa K, Miyake N, Yoshimura J, Okamura K, Niihori T, Saitsu H (2016). Human genetic variation database, a reference database of genetic variations in the Japanese population. J Hum Genet..

[25] Narahara M, Higasa K, Nakamura S, Tabara Y, Kawaguchi T, Ishii M (2014). Large-scale East-Asian eQTL mapping reveals novel candidate genes for LD mapping and the genomic landscape of transcriptional effects of sequence variants. PLoS One..

[26] Lee S, Seo J, Park J, Nam JY, Choi A, Ignatius JS (2017). Korean Variant Archive (KOVA): a reference database of genetic variations in the Korean population. Sci Rep..

[27] Picot IC, Ledroit M, Mockel L, Droin V, Daudon M, Zaidin M (2014). Adenine phosphoribosyltransferase deficiency: an under-recognized cause of urolithiasis and renal failure. J Nephrol Ther.

[28] Chen CJ, Schumacher HR (2009). Adenine phosphoribosyltransferase deficiency in a Chinese man with early-onset gout. J Rheumatol..

[29] Barbour B, Salameh P (2009). Consanguinity in Lebanon: prevalence, distribution and determinants. J Biosoc Sci..

[30] Ng SB, Buckingham KJ, Lee C, Bigham AW, Tabor HK, Dent KM (2010). Exome sequencing identifies the cause of a mendelian disorder. Nat Genet..

[31] Muona M, Berkovic SF, Dibbens LM, Oliver KL, Maljevic S, Bayly MA (2015). A recurrent de novo mutation in KCNC1 causes progressive myoclonus epilepsy. Nat Genet..

[32] McRae JF, Clayton S, Fitzgerald T, Kaplanis J, Prigmore E, Rajan D (2017). Prevalence and architecture of de novo mutations in developmental disorders. Nature..

[33] Boycott KM, Hartley T, Biesecker LG, Gibbs RA, Innes AM, Riess O (2019). A diagnosis for all rare genetic diseases: the horizon and the next frontiers. Cell..

