# Supplementary material for: Allele frequency of variants reported to cause adenine phosphoribosyltransferase deficiency
Source: Eur J Hum Genet. 2021 Mar 11;29(7):1061–70. doi: 10.1038/s41431-020-00805-6 (PMC8298615; doi:10.1038/s41431-020-00805-6)
Supplement: Supplementary file 1 — Supplementary_Tables_1_To_7_Clean [file 41431_2020_805_MOESM1_ESM.docx]

**Supplemental Tables**

**Supplemental Table 1.** Pathogenic *APRT* variants reported in patients with adenine phosphoribosyltransferase deficiency.

| **No.** | **Build 37** | **Build 38** | **rsID** | **Location** | **Base change** | **Amino acid change** | **Variant type** | **Homozygotes (n)** | **Compound heterozygotes (n)** | **Countryψ** |
| --- | --- | --- | --- | --- | --- | --- | --- | --- | --- | --- |
| 1 | 16:88876105_88878308? | 16:88809697_88811900? |  |  | c.-1_*1del | p.0 | Gross alteration | 0 | 1§ | Japan (1) |
| 2 | 16:88878307 | 16:88811899 | rs930107496 | Exon 1 | c.1A>G | p.(Met1?) | Start lost? | 0 | 6§ | Hungary (2), France (3) |
| 3 | 16:88878306 | 16:88811898 | rs1180937573 | Exon 1 | c.2T>C | p.(Met1?) | Start lost? | 1§ | 0 | India† |
| 4 | 16:88878305 | 16:88811897 | rs918734933 | Exon 1 | c.3G>A | p.(Met1?) | Start lost? | 2§ | 3§ | Turkey (4), UK†, India†,  Japan (5) |
| 5 | 16:88878285 | 16:88811877 | rs1261219212 | Exon 1 | c.23dup | p.(Val9Glyfs*2) | Frameshift | 0 | 1§ | USA† |
| 6 | 16:88878250 | 16:88811842 |  | Exon 1 | c.58C>T | p.(Pro20Ser) | Missense | 0§ | 1 | UK‡ |
| 7 | 16:88878067 | 16:88811659 | rs761838152 | Intron 1 | c.81-3C>G | p.? | Splice | 2§ | 1§ | USA‡, Italy† |
| 8 | 16:88878066 | 16:88811658 | rs751779314 | Intron 1 | c.81-2A>G | p.? | Splice | 1§ | 0 | France (3) |
| 9 | 16:88878063 | 16:88811655 |  | Exon 2 | c.82G>C | p.(Asp28His) | Missense | 1§ | 0 | Spain (6) |
| 10 | 16:88878061 | 16:88811653 |  | Exon 2 | c.84C>A | p.(Asp28Glu) | Missense | 0 | 1§ | Italy (7) |
| 11 | 16:88878047 | 16:88811639 |  | Exon 2 | c.98T>C | p.(Leu33Pro) | Missense | 0 | 1§ | Japan (8) |
| 12 | 16:88878026 | 16:88811618 |  | Exon 2 | c.119G>C | p.(Arg40Pro) | Missense | 1§ | 0 | Morocco (4) |
| 13 | 16:88877985 | 16:88811577 | rs752977102 | Exon 2 | c.160C>G | p.(His54Asp) | Missense | 0 | 2§ | USA (9), UK† |
| 14 | 16:88877964_88877965insA | 16:88811556_88811557insA |  | Exon 2 | c.180_181insT | p.(Ile61fs*49) | Frameshift | 1§ | 1§ | Greece (2), USA (2) |
| 15 | 16:88877936_88877961del | 16:88811528_88811553del |  | Exon 2/ Intron 2 | c.184_187+22del | p.? | Deletion | 0 | 1§ | Italy (7) |
| 16 | 16:88876967 | 16:88810559 |  | Intron 2 | c.188-3C>G | p.0 | Splice | 1§‡ | 0 | Italy (10) |
| 17 | 16:88876856_88877109del | 16:88810448_88810701del |  | Intron 2/ Exon 3 | c.188-145_296del | p.? | Indel | 3§ | 1§ | Austria (2), France (3), Italy (11, 12) |
| 18 | 16:88876964 | 16:88810556 |  | Exon 3 | c.188G>A | p.(Gly63Asp) | Missense | 6§ | 0 | France (3), Lebanon (4), Finland (Middle Eastern origin) (13), Australia (Lebanese) (14), USA† |
| 19 | 16:88876958 | 16:88810550 | rs104894506 | Exon 3 | c.194A>T | p.(Asp65Val) | Missense | 39§ | 2§ | Iceland (2), Britain (2), France (3),  Spain (4), Australia (15) |
| 20 | 16:88876953 | 16:88810545 | rs369681854 | Exon 3 | c.199C>T | p.(Arg67*) | Nonsense | 1§ | 1§ | France (3), India† |
| 21 | 16:88876952 | 16:88810544 | rs762509151 | Exon 3 | c.200G>A | p.(Arg67Gln) | Missense | 1 | 3§ | USA (2), Japan (16) and China (17) |
| 22 | 16:88876925 | 16:88810517 |  | Exon 3 | c.227C>T | p.(Ala76Val) | Missense | 0 | 1§ | India‡ |
| 23 | 16:88876902 | 16:88810494 | rs200392753 | Exon 3 | c.250G>A | p.(Val84Met) | Missense | 0 | 1 | Japan (16) |
| 24 | 16:88876893 | 16:88810485 | rs3169258 | Exon 3 | c.259C>T | p.(Arg87*) | Nonsense | 2§ | 1§ | Pakistan (2), USA†, UK‡ |
| 25 | 16:88876891_88876894dup | 16:88810483_88810486dup | rs281860265 | Exon 3 | c.258_261dup | p.(Lys88Profs*23) | Frameshift | 4§ | 1§ | Japan (1, 18) |
| 26 | 16:88876888 | 16:88810480 |  | Exon 3 | c.264G>T | p.(Lys88Asn) | Missense | 0 | 1§ | USA† |
| 27 | 16:88810458_88810464del | 16:88810458_88810464del | rs776240467 | Exon 3 | c.280_286del | p.(Gly94Leufs*41) | Frameshift | 2§ | 1§ | Turkey (19), Hungary (20) |
| 28 | 16:88876865_88876866del | 16:88810457_88810458del | rs563575862 | Exon 3 | c.286_287del | p.(Thr96Serfs*13) | Frameshift | 1§ | 0 | Portugal (4) |
| 29 | 16:88876864_88876865del | 16:88810454_88810455del | rs1437920638 | Exon 3 | c.289_290del | p.(Leu97Valfs*12) | Frameshift | 2§ | 5§ | France (3) |
| **Supplemental Table 1 (continued).** | | | | | | | | | | |
| **No.** | **Build 37** | **Build 38** | **rsID** | **Location** | **Base change** | **Amino acid change** | **Variant type** | **Homozygotes (n)** | **Compound heterozygotes (n)** | **Countryψ** |
| 30 | 16:88876858 | 16:88810450 | rs104894507 | Exon 3 | c.294G>A | p.(Trp98*) | Nonsense | 16§ | 10§ | Japan (16, 21) |
| 31 | 16:88876841 | 16:88810433 |  | Exon 3 | c.311A>G | p.(Glu104Gly) | Missense | 1§ | 0 | Senegal (4) |
| 32 | 16:88876829dup | 16:88810421dup | rs281860263 | Intron 3 | c.321+2dup | p.? | Splice | 2§ | 0 | Germany (22) |
| 33 | 16:88876549 | 16:88810141 | rs104894508 | Exon 4 | c.329T>C | p.(Leu110Pro) | Missense | 2 | 1§ | Canada (20) , France (3) |
| 34 | 16:88876544 | 16:88810136 | rs767177754 | Exon 4 | c.334A>T | p.(Ile112Phe) | Missense | 0 | 1§ | Bermuda (2) |
| 35 | 16:88876526 | 16:88810118 | rs370665100 | Exon 4 | c.352G>C | p.(Glu118Gln) | Missense | 0 | 2§ | France (3) |
| 36 | 16:88876519 | 16:88810111 | rs776948275 | Exon 4 | c.359G>T | p.(Gly120Val) | Missense | 1 | 0 | Spain (23) |
| 37 | 16:88876507 | 16:88810099 |  | Exon 4 | c.371T>G | p.(Val124Gly) | Missense | 3§ | 0 | France (3) |
| 38 | 16:88876498 | 16:88810090 |  | Exon 4 | c.380A>G | p.(Asp127Gly) | Missense | 1§ | 0 | UK (24) |
| 39 | 16:88876480 | 16:88810072 |  | Exon 4 | c.398G>A | p.(Gly133Asp) | Missense | 1 | 0 | Japan (16) |
| 40 | 16:88876477 | 16:88810069 |  | Intron 4 | c.400+1G>T | p.? | Splice | 1§ | 0 | Iraq (25) |
| 41 | 16:88876475 | 16:88810067 |  | Intron 4 | c.400+3A>T | p.? | Splice | 0 | 1§ | France (26) |
| 42 | 16:88876476dup | 16:88810068dup | rs745594160 | Intron 4 | c.400+2dup | p.(Ala108Glufs*3) | Indel | 20§ | 24§ | Italy (27), France (3), Poland (28), Germany (2), Austria (29), USA†, Belgium (2), Australia (15) |
| 43 | 16:88876489 | 16:88810081 |  | Exon 5 | c.389T>C | p.(Leu130Pro) | Missense | 0 | 1§ | France (26) |
| 44 | 16:88876242 | 16:88809834 | rs28999113 | Exon 5 | c.407T>C | p.(Met136Thr) | Missense | 90§ | 44§ | Japan (1, 30-41), USA† |
| 45 | 16:88876221 | 16:88809813 |  | Exon 5 | c.428T>C | p.(Leu143Pro) | Missense | 0 | 1 | France (3) |
| 46 | 16:88876210 | 16:88809802 | rs745872435 | Exon 5 | c.439C>T | p.(Gln147*) | Nonsense | 1§ | 0 | Italy (42) |
| 47 | 16:88876201 | 16:88809793 | rs281860266 | Exon 5 | c.448G>T | p.(Val150Phe) | Missense | 0 | 1§ | Germany (28) |
| 48 | 16:88876192 | 16:88809784 |  | Exon 5 | c.457T>C | p.(Cys153Arg) | Missense | 0 | 1§ | Bermuda (2) |
| 49 | 16:88876187_88876188del | 16:88809779_88809780del |  | Exon 5 | c.461_462del | p.(Val154Glufs*9) | Frameshift | 0 | 2 | China (17) |
| 50 | 16:88876175_88876177del | 16:88809767_88809769del |  | Exon 5 | c.472_474del | p.(Glu158del) | Deletion | 0 | 1§ | France (4) |
| 51 | 16:88876158 | 16:88809750 | rs768425517 | Exon 5 | c.491G>A | p.(Gly164Asp) | Missense | 0 | 1§ | France (43) |
| 52 | 16:88876139del | 16:88809731del |  | Exon 5 | c.510del | p.(Val171Tyrfs*82) | Deletion | 0 | 1§ | France (3) |
| 53 | 16:88876129del;  88876122_88876123del | 16:88809721del;  88809714_88809715del |  | Exon 5 | c.520del; 526_527del | p.? | Deletion | 0 | 1§ | France (26) |
| 54 | 16:88876126_88876128del | 16:88809718_88809720del | rs121912681 | Exon 5 | c.521_523del | p.(Phe174del) | Deletion | 3§ | 6§ | Belgium (44), USA†, France (3), UK‡ |
| 55 | 16:88876125 | 16:88809717 |  | Exon 5 | c.524C>T | p.(Ser175Phe) | Missense | 0 | 1§ | France (3) |
| 56 | 16:88876125_88876127del | 16:88809717_88809719del |  | Exon 5 | c.522_524del | p.(Ser175del) | Deletion | 0 | 1§ | USA† |
| 57 | 16:88876123 | 16:88809715 |  | Exon 5 | c.526C>T | p.(Leu176Phe) | Missense | 0 | 2§ | France (3) |
| 58 | 16:88876119_88876123del | 16:88809711_88809715del | rs755380873 | Exon 5 | c.526_530del | p.(Leu176Alafs*3) | Frameshift | 1§ | 0 | India (45) |
| 59 | 16:88876117 | 16:88809709 | rs1165408563 | Exon 5 | c.532C>T | p.(Gln178*) | Nonsense | 0 | 1§ | France (3) |
| 60 | 16:88876108 | 16:88809700 | rs758634272 | Exon 5 | c.541T>C | p.(*181Argext*?) | Nonsense | 0 | 1§ | France (4) |
| 61 | 16:88876107 | 16:88809699 | rs387906584 | Exon 5 | c.542G>C | p.(*181Serext*?) | Nonsense | 1§ | 0 | Japan (46) |
| 62 | 16:88876106 | 16:88809698 |  | Exon 5 | c.543A>T | p.(*181Cysext*?) | Nonsense | 2§ | 0 | UK (24) |

Reference sequence: NG_008013.1 (NM_000485.2).

§Enzyme function completely abolished *in vivo*.

†From the APRT deficiency Registry of the Rare Kidney Stone Consortium.

‡Personal communication.

ψNumbers in parentheses correspond to the source of case report in the reference list at the end of the document.

**Supplemental Table 2.** Compound heterozygous pathogenic *APRT* variants reported in patients with adenine phosphoribosyltransferase deficiency.

| No. | Base change | Amino acid change | Variant type | Country | Number of casesψ |
| --- | --- | --- | --- | --- | --- |
| 1 | c.-1_*1del; c.294G>A | p.0; p.(Trp98*) | Gross deletion; nonsense | Japan | 1 (1) |
| 2 | c.1A>G; c.280_286del | p.(Met1?); p.(Gly94Leufs*41) | Start lost?; frameshift | Hungary | 1 (2) |
| 3 | c.1A>G; c.352G>C | p.(Met1?); p.(Glu118Gln) | Start lost?; missense | France | 2 (3) |
| 4 | c.1A>G; c.400+2dup | p.(Met1?); p.(Ala108Glufs*3) | Start lost?; indel | France | 2 (3) |
| 5 | c.1A>G; c.491G>A | p.(Met1?); p.(Gly164Asp) | Start lost?; missense | France | 1 (3) |
| 6 | c.3G>A; c.160C>G | p.(Met1?); p.(His54Asp) | Start lost?; missense | UK | 1† |
| 7 | c.3G>A; c.227C>T | p.(Met1?); p.(Ala76Val) | Start lost?; missense | India | 1† |
| 8 | c.3G>A; c.407T>C | p.(Met1?); p.(Met136Thr) | Start lost?; missense | Japan | 1 (5) |
| 9 | c.23dup; c.522_524del | p.(Val9Glyfs*2); p.(Ser175del) | Frameshift; deletion | USA | 1† |
| 10 | c.58C>T; c.521_523del | p.(Pro20Ser); p.(Phe174del) | Missense; deletion | UK | 1‡ |
| 11 | c.81-3C>G; NA | p.?; NA | Splice; NA | Italy | 1† |
| 12 | c.84C>A; c.184_187+22del | p.(Asp28Glu); p.? | Missense; deletion | Italy | 1 (7) |
| 13 | c.98T>C; c.407T>C | p.(Leu33Pro); p.(Met136Thr) | Missense; missense | Japan | 1 (8) |
| 14 | c.160C>G; NA | p.(His54Asp); NA | Missense; NA | USA | 1 (9) |
| 15 | c.180_181insT; c.200G>A | p.(Ile61fs*49); p.(Arg67Gln) | Frameshift; missense | USA | 1 (2) |
| 16 | c.188-145_296del; c.400+2dup | p.?; p.(Ala108Glufs*3) | Indel; indel | Austria | 1 (2) |
| 17 | c.194A>T; c.329T>C | p.(Asp65Val); p.(Leu110Pro) | Missense; missense | France | 1 (3) |
| 18 | c.194A>T; c.400+2dup | p.(Asp65Val); p.(Ala108Glufs*3) | Missense; indel | Australia | 1 (15) |
| 19 | c.199C>T; c.400+2dup | p.(Arg67*); p.(Ala108Glufs*3) | Nonsense; indel | France | 1 (3) |
| 20 | c.200G>A; c.461_462del | p.(Arg67Gln); p.(Val154Glufs*9) | Missense; frameshift | China | 2 (17) |
| 21 | c.250G>A; c.294G>A | p.(Val84Met); p.(Trp98*) | Missense; nonsense | Japan | 1 (16) |
| 22 | c.264G>T; c.259C>T | p.(Lys88Asn); p.(Arg87*) | Missense; nonsense | USA | 1† |
| 23 | c.258_261dup; c.407T>C | p.(Lys88Profs*23); p.(Met136Thr) | Frameshift; missense | Japan | 1 (1) |
| 24 | c.289_290del; c.400+2dup | p.(Leu97Valfs*12); p.(Ala108Glufs*3) | Frameshift, indel | France | 4 (3) |
| 25 | c.289_290del; c.521_523del | p.(Leu97Valfs*12); p.(Phe174del) | Frameshift; deletion | France | 1 (3) |
| 26 | c.334A>T; c.457T>C | p.(Ile112Phe); p.(Cys153Arg) | Missense; missense | Bermuda | 1 (2) |
| 27 | c.400+3A>T; NA | p.?; NA | Splice; NA | France | 1 (26) |
| 28 | c.400+2dup; c.521_523del | p.(Ala108Glufs*3); p.(Phe174del) | Indel; deletion | Belgium | 2 (2) |
|  |  |  |  | France | 2 (3) |
| 29 | c.400+2dup; c.526C>T | p.(Ala108Glufs*3); p.(Leu176Phe) | Indel; missense | France | 2 (3) |
| 30 | c.400+2dup; c.428T>C | p.(Ala108Glufs*3); p.(Leu143Pro) | Indel; missense | France | 1 (3) |
| 31 | c.400+2dup; c.472_474del | p.(Ala108Glufs*3); p.(Glu158del) | Indel; deletion | France | 1 (3) |
| 32 | c.400+2dup; c.510del | p.(Ala108Glufs*3); p.(Val171Tyrfs*82) | Indel; deletion | France | 1 (3) |
| 33 | c.400+2dup; c.524C>T | p.(Ala108Glufs*3); p.(Ser175Phe) | Indel; missense | France | 1 (3) |
| 34 | c.400+2dup; NF | p.(Ala108Glufs*3); NF | Indel; NF | France | 3 (3) |
|  |  |  |  | Italy | 1 (27) |
| Supplemental Table 2 (continued). | | | | | |
| No. | **Base change** | **Amino acid change** | **Variant type** | **Country** | **Number of cases** |
| 35 | c.400+2dup; c.448G>T | p.(Ala108Glufs*3); p.(Val150Phe) | Indel; missense | Germany (Polish) | 1 (2) |
| 36 | c.389T>C; NA | p.(Leu130Pro); NA | Missense; NA | France | 1 (26) |
| 37 | c.407T>C; c.294G>A | p.(Met136Thr); p.(Trp98*) | Missense; nonsense | Japan | 8 (1) |
| 38 | c. 407T>C; NA | p.(Met136Thr); NA | Missense; NA | Japan | 33 (1, 30-31, 33, 37-41) |
| 39 | c.520del; 526_527del; NA | p.?; NA | Deletion; NA | France | 1 (26) |
| 40 | c.532C>T; NF | p.(Gln178*); NF | Nonsense; NF | France | 1 (3) |
| 41 | c.541T>C; NF | p.(*181Argext*?); NF | Nonsense; NF | France | 1 (4) |

Reference sequence: NG_008013.1 (NM_000485.2).

†From the APRT deficiency Registry of the Rare Kidney Stone Consortium.

‡Personal communication.

NA, not available; NF, not found.

ψNumbers in parentheses correspond to the source of case report in the reference list at the end of the document.

**Supplemental Table 3.** Analysis of splice site strength distributions for known pathogenic *APRT* variants using consensus sequence frequencies, the Maximum Entropy score and the varSEAK Online splice prediction tool.

| Build 38 | rsID | Location | Base change | Variant type | Frequencies of donor and acceptor base changes | Score alt (%)* | Score ref (%)* | Delta score (%) | MaxEntScan alt§ | Delta MaxEntScan | Class† | Splice site prediction | varSEAK results |
| --- | --- | --- | --- | --- | --- | --- | --- | --- | --- | --- | --- | --- | --- |
| 16:88811659 | rs761838152 | Intron 1 | c.81-3C>G | Splice | 0.8% | -49.68 | -10.66 | -39.02 | -5.76 | -11.42 | 4 (likely splicing effect) | 3' acceptor splice site prediction: Exon skipping. Likely loss of function for authentic splice site. | [Https://varseak.bio/ssp.php?Gene=APRT&transcript=NM_000485.3&variant=&hgvs=c.81-3C%3EG](https://varseak.bio/ssp.php?gene=APRT&transcript=NM_000485.3&variant=&hgvs=c.81-3C%3EG) |
| 16:88811658 | rs751779314 | Intron 1 | c.81-2A>G | Splice | 0.1% | < No AG > | -10.66 | NA | -2.29 | -7.96 | 5 (splicing effect) | 3' acceptor splice site prediction: Exon skipping. No AG. Loss of function for authentic splice site. | [Https://varseak.bio/ssp.php?Gene=APRT&transcript=NM_000485.3&variant=&hgvs=c.81-2A%3EG](https://varseak.bio/ssp.php?gene=APRT&transcript=NM_000485.3&variant=&hgvs=c.81-2A%3EG) |
| 16:88810559 |  | Intron 2 | c.188-3C>G | Splice | 0.8% | -40.56 | 20.75 | -61.31 | -0.51 | -9.75 | 5 (splicing effect) | 3' acceptor splice site prediction: Use of cryptic site 44 nucleotides downstream of 3' splice site. Loss of function for authentic splice site. | [Https://varseak.bio/ssp.php?Gene=APRT&transcript=NM_000485.3&variant=&hgvs=c.188-3C%3EG](https://varseak.bio/ssp.php?gene=APRT&transcript=NM_000485.3&variant=&hgvs=c.188-3C%3EG) |
| 16:88810421dup | rs281860263 | Intron 2 | c.321+2dup | Splice |  | -18.01 | 86.39 | -104.4 | 3.39 | -7.18 | 5 (splicing effect) | 5' donor splice site prediction: Loss of function for authentic splice site. Exon skipping. Strong decrease of score for authentic splice site. | [Https://varseak.bio/ssp.php?Gene=APRT&transcript=NM_000485.3&variant=&hgvs=c.321%2b2dup](https://varseak.bio/ssp.php?gene=APRT&transcript=NM_000485.3&variant=&hgvs=c.321%2B2dup) |
| 16:88810069 |  | Intron 4 | c.400+1G>T | Splice | 0.1% | < No GT > | 46.89 | NA | -0.14 | -8.50 | 5 (splicing effect) | 5' donor splice site prediction: Loss of function for authentic splice site. Exon skipping. No GT. Loss of function for authentic splice site. | [Https://varseak.bio/ssp.php?Gene=APRT&transcript=NM_000485.3&variant=&hgvs=c.400%2B1G%3ET](https://varseak.bio/ssp.php?gene=APRT&transcript=NM_000485.3&variant=&hgvs=c.400%2B1G%3ET) |
| 16:88810067 |  | Intron 4 | c.400+3A>T | Splice | 3.4% | -66.14 | 46.89 | -113.03 | 0.95 | -7.42 | 5 (splicing effect) | 5' donor splice site prediction: Loss of function for authentic splice site. Exon skipping. Strong decrease of score for authentic splice site. | [Https://varseak.bio/ssp.php?Gene=APRT&transcript=NM_000485.3&hgvs=c.400%2B3A%3ET](https://varseak.bio/ssp.php?gene=APRT&transcript=NM_000485.3&hgvs=c.400%2B3A%3ET) |
| 16:88810068dup | rs745594160 | Intron 4 | c.400+2dup | Indel |  | -44.33 | 46.89 | -91.22 | -4.78 | -13.15 | 5 (splicing effect) | 5' donor splice site prediction: Loss of function for authentic splice site. Exon skipping. Strong decrease of score for authentic splice site. | [Https://varseak.bio/ssp.php?Gene=APRT&transcript=NM_000485.3&variant=&hgvs=c.400%2b2dupt](https://varseak.bio/ssp.php?gene=APRT&transcript=NM_000485.3&variant=&hgvs=c.400%2B2dupT) |
| 16:88811528  _88811553del |  | Exon 2/ intron 2 | c.184_187+22del | Deletion |  | NA | NA | NA | -8.61 | -11.53 | NA‡ | NA | NA |
| 16:88810448  _88810701del |  | Intron 2/ exon 3 | c.188-145_296del | Indel |  | NA | NA | NA | -7.93 | -16.56 | NA‡ | NA | NA |

Reference sequence: NG_008013.1 (NM_000485.2).

*varSEAK score: Indicates the likelihood that a variant is predicted to be a functional splice site (positive value) or not a functional splice site (negative value), ranging from -100% to +100%. For splice sites that are equally likely to work and not to work, the score is 0%.

§MaxEntScan: Splice site model that assigns a log odds ratio (MaxENT score) to a given sequence. The higher the score, the higher the probability that the sequence is a true splice site. From Yeo and Burge, 2004 (http://web.mit.edu/~fyfer/tmp-public/yeo-2004-jcompiol-maxent.pdf).

†Classes given by the online varSEAK tool: Class 1. No splicing effect; Class 2. Likely no splicing effect; Class 3. Unknown splicing effect; Class 4. Likely splicing effect; Class 5. Splicing effect.

‡Variant not suitable for the varSEAK Online splice prediction tool. The delta MaxEntScan score was therefore manually curated. These larger deletions are expected to result in abnormal splicing as suggested by the low delta MaxEntScan scores.

Abbreviation: NA, not applicable.

**Supplemental Table 4.** Known pathogenic *APRT* variants found in the 100.000 Genomes Project database.

| **No.** | **Position**  **(Build 37)** | **Position**  **(Build 38)** | **rsID** | **Location** | **Base change** | **Amino acid change** | **Variant type** | **Allele count** | **Allele number** | **MAF (%)** |
| --- | --- | --- | --- | --- | --- | --- | --- | --- | --- | --- |
| **1** | 16:88876958 | 16:88810550 | rs104894506 | Exon 3 | c.194A>T | p.(Asp65Val) | Missense | 1 | 127,474 | 0.001 |
| **2** | 16:88876476dup | 16:88810068dup | rs745594160 | Intron 4 | c.400+2dup | p.(Ala108Glufs*3) | Indel | 4 | 127,474 | 0.003 |
| **3** | 16:88876526 | 16:88810118 | rs370665100 | Exon 4 | c.352G>C | p.(Glu118Gln) | Missense | 1 | 127,474 | 0.001 |
| **4** | 16:88877985 | 16:88811577 | rs752977102 | Exon 2 | c.160C>G | p.(His54Asp) | Missense | 2 | 127,474 | 0.002 |
| **5** | 16:88878305 | 16:88811897 | rs918734933 | Exon 1 | c.3C>T | p.(Met1?) | Nonsense | 2 | 127,474 | 0.002 |
| **6** | 16:88876126_88876128del | 16:88809718_88809720del | rs121912681 | Exon 5 | c.521_523del | p.(Phe174del) | Deletion | 1 | 127,474 | 0.001 |

Reference sequence: NG_008013.1 (NM_000485.2).

Abbreviation: MAF, minor allele frequency.

**Supplemental Table 5.** Known pathogenic *APRT* variants found in the Genome Aggregation Database (gnomAD) (v2.1.1).

| **No.** | **Position**  **(Build 37)** | **Position**  **(Build 38)** | **rsID** | **Location** | **Base change** | **Amino acid change** | **Variant type** | **Allele count** | **MAF (%)** |
| --- | --- | --- | --- | --- | --- | --- | --- | --- | --- |
| **1** | 16:88876476dup | 16:88810068dup | rs745594160 | Intron 4 | c.400+2dup | p.(Ala108Glufs*3) | Indel | 30 | 0.010630 |
| **2** | 16:88876126_88876128del | 16:88809718_88809720del | rs121912681 | Exon 5 | c.521_523del | p.(Phe174del) | Indel | 10 | 0.006977 |
| **3** | 16:88876902 | 16:88810494 | rs200392753 | Exon 4 | c.250G>A | p.(Val84Met) | Missense | 9 | 0.003224 |
| **4** | 16:88877985 | 16:88811577 | rs752977102 | Exon 3 | c.160C>G | p.(His54Asp) | Missense | 7 | 0.002806 |
| **5** | 16:88876858 | 16:88810450 | rs104894507 | Exon 3 | c.294G>A | p.(Trp98*) | Nonsense | 6 | 0.002423 |
| **6** | 16:88876158 | 16:88809750 | rs768425517 | Exon 3 | c.491G>A | p.(Gly164Asp) | Missense | 4 | 0.001596 |
| **7** | 16:88876201 | 16:88809793 | rs281860266 | Exon 3 | c.448G>T | p.(Val150Phe) | Missense | 4 | 0.001600 |
| **8** | 16:88876526 | 16:88810118 | rs370665100 | Exon 3 | c.352G>C | p.(Glu118Gln) | Missense | 4 | 0.001418 |
| **9** | 16:88876544 | 16:88810136 | rs767177754 | Exon 5 | c.334A>T | p.(Ile112Phe) | Missense | 3 | 0.001064 |
| **10** | 16:88876862_88876865del | 16:88810454_88810457del | rs1437920638 | Exon 5 | c.289_290del | p.(Leu97Valfs*12) | Frameshift | 3 | 0.001075 |
| **11** | 16:88876893 | 16:88810485 | rs3169258 | Exon 3 | c.259C>T | p.(Arg87*) | Nonsense | 3 | 0.001210 |
| **12** | 16:88878067 | 16:88811659 | rs761838152 | Intron 1 | c.81-3C>G | p.? | Splice | 3 | 0.001521 |
| **13** | 16:88878305 | 16:88811897 | rs918734933 | Exon 1 | c.3G>A | p.(Met1?) | Nonsense | 3 | 0.002043 |
| **14** | 16:88876549 | 16:88810141 | rs104894508 | Exon 4 | c.329T>C | p.(Leu110Pro) | Missense | 2 | 0.000799 |
| **15** | 16:88876953 | 16:88810545 | rs369681854 | Exon 3 | c.199C>T | p.(Arg67*) | Nonsense | 2 | 0.000818 |
| **16** | 16:88876953 | 16:88810545 | rs766646831 | Intron 2 | c.188-3C>G | p.0 | Splice | 2 | 0.000827 |
| **17** | 16:88878285 | 16:88811877 | rs1261219212 | Exon 1 | c.23dup | p.(Val9Glyfs*2) | Frameshift | 2 | 0.001017 |
| **18** | 16:88878307 | 16:88811899 | rs930107496 | Exon 1 | c.1A>G | p.(Met1?) | Nonsense | 2 | 0.001378 |
| **19** | 16:88876107 | 16:88809699 | rs387906584 | Exon 5 | c.542G>C | p.(*181Serext*?) | Nonsense | 1 | 0.003186 |
| **20** | 16:88876108 | 16:88809700 | rs758634272 | Exon 5 | c.541T>C | p.(*181Argext*?) | Nonsense | 1 | 0.000399 |
| **21** | 16:88876117 | 16:88809709 | rs1165408563 | Exon 5 | c.532C>T | p.(Gln178*) | Nonsense | 1 | 0.000399 |
| **22** | 16:88876119_88876123del | 16:88809711_88809715del | rs755380873 | Exon 5 | c.526_530del | p.(Leu176Alafs*3) | Frameshift | 1 | 0.000399 |
| **23** | 16:88876210 | 16:88809802 | rs745872435 | Exon 5 | c.439C>T | p.(Gln147*) | Nonsense | 1 | 0.000400 |
| **24** | 16:88876242 | 16:88809834 | rs28999113 | Exon 5 | c.407T>C | p.(Met136Thr) | Missense | 1 | 0.000403 |
| **25** | 16:88876865_88876866del | 16:88810457_88810458del | rs563575862 | Exon 3 | c.286_287del | p.(Thr96Serfs*13) | Frameshift | 1 | 0.000404 |
| **26** | 16:88876888 | 16:88810480 | rs138781159 | Exon 3 | c.264G>T | p.(Lys88Asn) | Missense | 1 | 0.000403 |
| **27** | 16:88876952 | 16:88810544 | rs762509151 | Exon 3 | c.200G>A | p.(Arg67Gln) | Missense | 1 | 0.000408 |
| **28** | 16:88878066 | 16:88811658 | rs751779314 | Intron 1 | c.81-2A>G | p.? | Splice | 1 | 0.000497 |

Reference sequence: NG_008013.1 (NM_000485.2).

Abbreviation: MAF, minor allele frequency.

**Supplemental Table 6.** Known pathogenic *APRT* variants found in the UK Biobank.

| **No.** | **Position**  **(Build 37)** | **Position**  **(Build 38)** | **rsID** | **Location** | **Base change** | **Amino acid change** | **Variant type** | **Allele count** | **Allele number** | **MAF (%)** |
| --- | --- | --- | --- | --- | --- | --- | --- | --- | --- | --- |
| **1** | 16:88877985 | 16:88811577 | rs752977102 | Exon 2 | c.160C>G | p.(His54Asp) | Missense | 24 | 100,000 | 0.024 |
| **2** | 16:88876476dup | 16:88810068dup | rs745594160 | Intron 4 | c.400+2dup | p.(Ala108Glufs*3) | Indel | 22 | 100,000 | 0.022 |
| **3** | 16:88876526 | 16:88810118 | rs370665100 | Exon 4 | c.352G>C | p.(Glu118Gln) | Missense | 15 | 100,000 | 0.015 |
| **4** | 16:88876126_88876128del | 16:88809718_88809720del | rs121912681 | Exon 5 | c.521_523del | p.(Phe174del) | Deletion | 3 | 100,000 | 0.003 |
| **5** | 16:88876549 | 16:88810141 | rs104894508 | Exon 4 | c.329T>C | p.(Leu110Pro) | Missense | 3 | 100,000 | 0.003 |
| **6** | 16:88876953 | 16:88810545 | rs369681854 | Exon 3 | c.199C>T | p.(Arg67*) | Nonsense | 2 | 100,000 | 0.002 |
| **7** | 16:88876108 | 16:88809700 | rs758634272 | Exon 5 | c.541T>C | p.(*181Argext*?) | Nonsense | 1 | 100,000 | 0.001 |
| **8** | 16:88876210 | 16:88809802 | rs745872435 | Exon 5 | c.439C>T | p.(Gln147*) | Nonsense | 1 | 100,000 | 0.001 |

Reference sequence: NG_008013.1 (NM_000485.2).

Abbreviation: MAF, minor allele frequency.

**Supplemental Table 7.** *APRT* variants of uncertain significance found in the Genome Aggregation Database (gnomAD) (v2.1.1).

| **No.** | **Position**  **(Build 37)** | **Position**  **(Build 38)** | **rsID** | **Location** | **Base change** | **Amino acid change** | **Variant type** | **Allele count** | **Allele number** | **MAF (%)** |
| --- | --- | --- | --- | --- | --- | --- | --- | --- | --- | --- |
| **1** | 16:88876886 | 16:88810478 | rs150156607 | Exon 3 | c.266G>A | p.(Arg89Gln) | Missense | 237 | 279,314 | 0.06 |
| **2** | 16:88877960 | 16:88811552 | rs201579274 | Exon 3 | c.185C>T | p.(Ala62Val) | Missense | 140 | 236,708 | 0.06 |
| **3** | 16:88876516 | 16:88810108 | rs8191494 | Exon 4 | c.362A>G | p.(Gln121Arg) | Missense | 130 | 282,280 | 0.05 |
| **4** | 16:88876502 | 16:88810094 | rs75205792 | Exon 4 | c.376G>C | p.(Val126Met) | Missense | 58 | 282,292 | 0.02 |
| **5** | 16:88876532 | 16:88810124 | rs201944035 | Exon 4 | c.346G>A | p.(Ala116Thr) | Missense | 58 | 282,044 | 0.02 |
| **6** | 16:88876537 | 16:88810129 | rs151240811 | Exon 4 | c.341A>C | p.(Lys114Thr) | Missense | 45 | 282,040 | 0.02 |
| **7** | 16:88876505 | 16:88810097 | rs376629164 | Exon 4 | c.373G>A | p.(Val125Ile) | Missense | 34 | 282,286 | 0.01 |
| **8** | 16:88876851 | 16:88810443 | rs551418842 | Exon 3 | c.301T>C | p.(Tyr101His) | Missense | 34 | 247,540 | 0.01 |
| **9** | 16:88876836 | 16:88810428 | rs780098835 | Exon 3 | c.316G>A | p.(Gly106Arg) | Missense | 23 | 278,010 | 0.008 |
| **10** | 16:88877970 | 16:88811562 | rs768268700 | Exon 3 | c.175G>A | p.(Asp59Asn) | Missense | 21 | 242,620 | 0.009 |

Reference sequence: NG_008013.1 (NM_000485.2).

**References**

1. Kamatani N, Hakoda M, Otsuka S, Yoshikawa H, Kashiwazaki S. Only three mutations account for almost all defective alleles causing adenine phosphoribosyltransferase deficiency in Japanese patients. J Clin Invest. 1992;90(1):130-5.

2. Sahota A, Tischfield J, Kamatani N, Simmonds H. Adenine phosphoribosyltransferase deficiency and 2,8-dihydroxyadenine lithiasis. In: Scriver CR, Beaudet AL, Sly WS, Valle D, Vogelstein B, Childs B, eds. The Metabolic and Molecular Bases of Inherited Disease. Vol 1. 8th ed. New York, NY: McGraw-Hill; 2001:2571-84.

3. Ceballos-Picot I, Ledroit M, Mockel L, Droin V, Daudon M, Zaidin M, et al. Adenine phosphoribosyltransferase deficiency: an under-recognized cause of urolithiasis and renal failure. J Nephrol Ther. 2014;04(04).

4. Bollee G, Dollinger C, Boutaud L, Guillemot D, Bensman A, Harambat J, et al. Phenotype and genotype characterization of adenine phosphoribosyltransferase deficiency. J Am Soc Nephrol. 2010;21(4):679-88.

5. Ikeda H, Watanabe T, Fujimoto Y, Yamamoto S, Hosaki I, Isoyama K, et al. [The first case of adenine phosphoribosyltransferase deficiency with APRT*Q0 (M1I) mutation in Japan]. Hinyokika Kiyo. 2012;58(7):15-9. Japanese.

6. Rodriguez-Borja E, Corchon-Peyrallo A, Quinones-Torrelo C, Ramos-Tomas C. Acute kidney failure and discrepant values of urinary proteins: When the case is not "crystal clear". Clin Biochem. 2019;67:60-2.

7. Marra G, Vercelloni PG, Edefonti A, Manzoni G, Pavesi MA, Fogazzi GB, et al. Adenine phosphoribosyltransferase deficiency: an underdiagnosed cause of lithiasis and renal failure. JIMD Rep. 2012;5:45-8.

8. Nozue H, Kamoda T, Saitoh H, Ichikawa K, Taniguchi A. A Japanese boy with adenine phosphoribosyltransferase (APRT) deficiency caused by compound heterozygosity including a novel missense mutation in APRT gene. Acta Paediatr. 2011;100(12):e285-8.

9. Cochran B, Kovacikova T, Hodanova K, Zivna M, Hnizda A, Niehaus AG, et al. Chronic tubulointerstitial kidney disease in untreated adenine phosphoribosyl transferase (APRT) deficiency: A case report. Clin Nephrol. 2018;90(4):296-301.

10. Micheli V, Massarino F, Jacomelli G, Bertelli M, Corradi MR, Guerrini A, et al. Adenine phosphoribosyltransferase (APRT) deficiency: a new genetic mutation with early recurrent renal stone disease in kidney transplantation. NDT Plus. 2010;3(5):436-8.

11. Di Pietro V, Perruzza I, Amorini AM, Balducci A, Ceccarelli L, Lazzarino G, et al. Clinical, biochemical and molecular diagnosis of a compound homozygote for the 254 bp deletion-8 bp insertion of the APRT gene suffering from severe renal failure. Clin Biochem. 2007;40(1-2):73-80.

12. Perruzza I, Di Pietro V, Tavazzi B, Lazzarino G, Gamberini M, Barsotti P, et al. Is adenine phophorybosiltransferase deficiency a still underdiagnosed cause of urolithiasis and chronic renal failure? A report of two cases in a family with an uncommon novel mutation. NDT Plus. 2008;1(5):292-5.

13. Kaartinen K, Hemmila U, Salmela K, Raisanen-Sokolowski A, Kouri T, Makela S. Adenine phosphoribosyltransferase deficiency as a rare cause of renal allograft dysfunction. J Am Soc Nephrol. 2014;25(4):671-4.

14. Li J, Shingde M, Nankivell BJ, Tchan MC, Bose B, Chapman JR, et al. Adenine phosphoribosyltransferase deficiency: a potentially reversible cause of CKD. Kidney Int Rep. 2019; 4(8):1161-70.

15. Huq A, Nand K, Juneja R, Winship I. APRT deficiency: the need for early diagnosis. BMJ Case Rep. 2018;2018:bcr2018225742.

16. Taniguchi A, Tsuchida S, Kuno S, Mita M, Machida T, Ioritani N, et al. Identification of two novel mutations in adenine phosphoribosyltransferase gene in patients with 2,8-dihydroxyadenine urolithiasis. Nucleosides Nucleotides Nucleic Acids. 2004;23(8-9):1141-5.

17. Lau NKC, Ng SKW, Chan IHS, Ng KS, Kwok JSS. Urinary bladder stone due to adenine phosphoribosyltransferase deficiency: first genetically confirmed case in a Chinese patient. Pathology 2019;51(5):557-61.

18. Higashimoto H, Ouchi A, Kawaguchi R. Detection of the three common mutations of adeninephosphoribosyltransferase deficiency among Japanese. Clin Chim Acta. 1995;234(1-2):1-10.

19. Bye S, Mallmann R, Duley J, Simmonds HA, Chen J, Tischfield JA, et al. Identification of a 7-basepair deletion in the adenine phosphoribosyltransferase gene as a cause of 2,8-dihydroxyadenine urolithiasis. Clin Investig. 1994;72(7):550-3.

20. Sahota A, Chen J, Bye S, Jaing J, Berenyi M, Fekete G, et al. Occurrence of a missense mutation in one allele and a seven basepair deletion in the other allele in a patient with adenine phosphoribosyltransferase deficiency. Hum Mutat. 1994;3(3):315-7.

21. Nanmoku K, Kurosawa A, Shinzato T, Shimizu T, Kimura T, Yagisawa T. Febuxostat for the prevention of recurrent 2,8-dihydroxyadenine nephropathy due to adenine phosphoribosyltransferase deficiency following kidney transplantation. Intern Med. 2017;56(11):1387-91.

22. Gathof BS, Sahota A, Gresser U, Chen J, Stambrook PJ, Tischfield JA, et al. Identification of a splice mutation at the adenine phosphoribosyltransferase locus in a German family. Klin Wochenschr. 1990;69(24):1152-5.

23. Jimenez Herrero MC, Petkov Stoyanov V, Gutierrez Sanchez MJ, Martin Navarro JA. Litiasis due to 2,8-dihydroxyadenine, usefulness of the genetic study. Nefrologia. 2019;39(2):206-7.

24. Balasubramaniam GS, Arenas-Hernandez M, Escuredo E, Fairbanks L, Marinaki T, Mapplebeck S, et al. Adenine phosphoribosyltransferase deficiency in the United Kingdom: two novel mutations and a cross-sectional survey. Clin Kidney J. 2016;9(6):800-6.

25. Chen J, Sahota A, Martin GF, Hakoda M, Kamatani N, Stambrook PJ, et al. Analysis of germline and in vivo somatic mutations in the human adenine phosphoribosyltransferase gene: mutational hot spots at the intron 4 splice donor site and at codon 87. Mutat Res. 1993;287(2):217-25.

26. Ceballos-Picot I, Daudon M, Harambat J, Bensman A, Knebelmann B, Bollée G. 2,8-Dihydroxyadenine urolithiasis: a not so rare inborn error of purine metabolism. Nucleosides Nucleotides Nucleic Acids. 2014;33(4-6):241-52.

27. Stratta P, Fogazzi GB, Canavese C, Airoldi A, Fenoglio R, Bozzola C, et al. Decreased kidney function and crystal deposition in the tubules after kidney transplant. Am J Kidney Dis. 2010;56(3):585-90.

28. Deng L, Yang M, Frund S, Wessel T, De Abreu RA, Tischfield JA, et al. 2,8-Dihydroxyadenine urolithiasis in a patient with considerable residual adenine phosphoribosyltransferase activity in cell extracts but with mutations in both copies of APRT. Mol Genet Metab. 2001;72(3):260-4.

29. Menardi C, Schneider R, Neuschmid-Kaspar F, Klocker H, Hirsch-Kauffmann M, Auer B, et al. Human APRT deficiency: indication for multiple origins of the most common Caucasian mutation and detection of a novel type of mutation involving intrastrand-templated repair. Hum Mutat. 1997;10(3):251-5.

30. Kamatani N, Terai C, Kim SY, Chen CL, Yamanaka H, Hakoda M, et al. The origin of the most common mutation of adenine phosphoribosyltransferase among Japanese goes back to a prehistoric era. Hum Genet. 1996;98(5):596-600.

31. Yamaguchi S, Haba T, Koike H. [A case report of 2,8-dihydroxyadenine stone]. Hinyokika Kiyo. 2015;61(7):279-83. Japanese.

32. Katsuoka Y, Miyakita H, Shiramizu M, Iwagaki H, Ikeda T. [2,8-Dihydroxyadenine urolithiasis due to partial deficit in adenine phosphoribosyltransferase: a case report]. Hinyokika Kiyo. 1992;38(5):573-7. Japanese.

33. Ikeda H, Watanabe T, Toyama D, Isoyama K. Use of LightCycler mutation analysis to detect type II adenine phosphoribosyltransferase deficiency in two patients with 2,8-dihydroxyadeninuria. CEN Case Rep. 2016;5(1):34-9.

33. Tanemoto M, Takeuchi Y, Mishima E, Suzuki T, Abe T, Ito S. Urinary stones resembling uric acid stones. NDT Plus. 2010;3(3):318-9.

34. Oyake C, Ikeda H, Fuyama M, Watanabe T, Isoyama K. Minimum allopurinol dose for adenine phosphoribosyl transferase deficiency. Pediatr Int. 2017;59(10):1097-8.

36. Kitami K, Kinoshita Y, Hosaka M. [A case of 2,8-dihydroxyadenine stone with a partial deficiency of adenine phosphoribosyltransferase]. Hinyokika Kiyo. 1990;36(1):73-6. Japanese.

37. Iwaki T, Kusaka T, Ohashi I, Nishida T, Imai T, Itoh S. Two families with compound heterozygosity for adenine phosphoribosyltransferase deficiency. Pediatr Nephrol. 2010;25(6):1173-6.

38. Ohne T, Fujito A, Koga K, Imaide Y, Uchida M. [2,8-dihydroxyadenine urolithiasis due to partial deficiency of adenine phosphoribosyltransferase: a case report]. Hinyokika Kiyo. 1998;44(10):725-8. Japanese.

39. Shiba M, Shimizu K, Takatera H. [2,8-dihydoroxyadenine (DHA) urolithiasis: a case report]. Hinyokika Kiyo. 2003;49(8):497-9. Japanese.

40. Takeuchi H, Kaneko Y, Fujita J, Yoshida O. A case of a compound heterozygote for adenine phosphoribosyltransferase deficiency (APRT*J/APRT*Q0) leading to 2,8-dihydroxyadenine urolithiasis: review of the reported cases with 2,8-dihydroxyadenine stones in Japan. J Urol. 1993;149(4):824-6.

41. Nakanishi S, Saito R, Mizuno K, Matsuoka T, Kita Y, Asai S, et al. [A case of bilateral renal calculi in a 1-year-old female with adenine phosphoribosyl transferase partial deficiency]. Hinyokika Kiyo. 2011;57(10):551-4. Japanese.

42. Valaperta R, Rizzo V, Lombardi F, Verdelli C, Piccoli M, Ghiroldi A, et al. Adenine phosphoribosyltransferase (APRT) deficiency: identification of a novel nonsense mutation. BMC Nephrol. 2014;15:102.

43. Harambat J, Bollee G, Daudon M, Ceballos-Picot I, Bensman A; APRT Study Group. Adenine phosphoribosyltransferase deficiency in children. Pediatr Nephrol. 2012;27(4):571-9.

44. Hidaka Y, Palella TD, O'Toole TE, Tarle SA, Kelley WN. Human adenine phosphoribosyltransferase. Identification of allelic mutations at the nucleotide level as a cause of complete deficiency of the enzyme. J Clin Invest. 1987;80(5):1409-15.

45. Ceballos-Picot I, Saha A, Arora N, Kapoor K, Kaur M, Dhull RS, et al. Adenine phosphoribosyltransferase deficiency due to novel mutation. Kidney Int Rep. 2019;4(4):624-8.

46. Taniguchi A, Hakoda M, Yamanaka H, Terai C, Hikiji K, Kawaguchi R, et al. A germline mutation abolishing the original stop codon of the human adenine phosphoribosyltransferase (APRT) gene leads to complete loss of the enzyme protein. Hum Genet. 1998;102(2):197-202.
